# Impact of Light Intensity on Antioxidant Activity of Tropical Microalgae

**DOI:** 10.3390/md18020122

**Published:** 2020-02-18

**Authors:** Noémie Coulombier, Elodie Nicolau, Loïc Le Déan, Cyril Antheaume, Thierry Jauffrais, Nicolas Lebouvier

**Affiliations:** 1 ADECAL Technopole, 1 bis rue Berthelot, 98846 Noumea, New Caledonia; 2 Ifremer, RBE/BRM/PBA, Rue de l’île d’Yeu, 44311 Nantes, France; Elodie.Nicolau@ifremer.fr; 3 Ifremer, UMR 9220 ENTROPIE, RBE/LEAD, 101 Promenade Roger Laroque, 98897 Noumea, New Caledonia; Loic.Le.Dean@ifremer.fr (L.L.D.); Thierry.Jauffrais@ifremer.fr (T.J.); 4 ISEA, EA7484, Université de Nouvelle Calédonie, Campus de Nouville, 98851 Nouméa, New Caledonia; antheaume@unistra.fr (C.A.); nicolas.lebouvier@univ-nc.nc (N.L.)

**Keywords:** *nephroselmis*, light intensity, in vitro antioxidant activity, siphonaxanthin, carotenoid, bioactive compounds

## Abstract

Twelve microalgae species isolated in tropical lagoons of New Caledonia were screened as a new source of antioxidants. Microalgae were cultivated at two light intensities to investigate their influence on antioxidant capacity. To assess antioxidant property of microalgae extracts, four assays with different modes of action were used: 1,1-diphenyl-2-picrylhydrazyl (DPPH), 2,2’-azino-bis (3-éthylbenzothiazoline-6-sulphonique) (ABTS), oxygen radical absorbance capacity (ORAC), and thiobabituric acid reactive substances (TBARS). This screening was coupled to pigment analysis to link antioxidant activity and carotenoid content. The results showed that none of the microalgae studied can scavenge DPPH and ABTS radicals, but *Chaetoceros* sp., *Nephroselmis* sp., and *Nitzschia* A sp. have the capacity to scavenge peroxyl radical (ORAC) and *Tetraselmis* sp., *Nitzschia* A sp., and *Nephroselmis* sp. can inhibit lipid peroxidation (TBARS). Carotenoid composition is typical of the studied microalgae and highlight the siphonaxanthin, detected in *Nephroselmis* sp., as a pigment of interest. It was found that xanthophylls were the major contributors to the peroxyl radical scavenging capacity measured with ORAC assay, but there was no link between carotenoids and inhibition of lipid peroxidation measured with TBARS assay. In addition, the results showed that light intensity has a strong influence on antioxidant capacity of microalgae: Overall, antioxidant activities measured with ORAC assay are better in high light intensity whereas antioxidant activities measured with TBARS assay are better in low light intensity. It suggests that different antioxidant compounds production is related to light intensity.

## 1. Introduction

In the last decade, the demand has increased for sustainable sources of natural antioxidants for nutritional, cosmetic, and pharmaceutical applications as an alternative to controversial synthetic antioxidants. Most natural antioxidants available on the market derive from terrestrial plants [1], but new antioxidants from marine origin are getting attention [2,3,4]. Microalgae are a promising source for natural antioxidant products [5,6], as their productivity is greater than terrestrial plant [7], culture conditions could be controlled, and marine microalgae production at a commercial scale does not compete with agriculture for freshwater access and arable land. In addition, to be adapted to a large range of environments, microalgae produce a large diversity of secondary metabolites [8,9]. This exceptional chemodiversity is being explored and is a promising source of antioxidant [10,11,12,13,14,15], as only few species have been investigated among the thousands described. To highlight the full potential of microalgae, identifications of new high producing strains and new compounds are needed. It is thus necessary to identify new strains with high productivity and/or new compounds of interest.

The production of secondary metabolites by microalgae is modulated by environmental conditions [16,17,18,19]. In response to abiotic stresses (i.e., high light, UV, salinity, temperature, metal concentration, or nutrient starvation), through photosynthesis and aerobic metabolism microalgae produce reactive oxygen species (ROS) which can be toxic and cause cell damages. Microalgae have developed defense strategies. One of them is the synthesis of an heterogeneous group of molecules which have the ability to delay, prevent, or remove oxidative damage to the cell [20]. It includes enzymes (e.g., superoxide dismutase and catalase) and non-enzymatic molecules such as carotenoids, phenolic acids, or vitamins C and E [21,22,23] that are present in high concentration in some species [24]. Carotenoids protect the cell against oxidative stress by dissipating excess of energy through the xanthophyll cycle [25,26,27] and by scavenging ROS, mainly singlet oxygen and peroxyl radical [28,29,30]. In an aquatic environment and especially in tropical areas, microalgae are submitted to strong light variation and have to quickly adapt to light excess or limitation. The effect of light on antioxidants production, especially carotenoids, is known to be complex and species specific [31,32,33,34,35,36]. While many studies focus on the effect of light on specific antioxidant molecules, investigations about its effect on global antioxidant activity of microalgae are scarce. However, nutraceuticals or aquaculture preparations often use the whole biomass or crude algal extract, with no purification of molecules of interest.

In this study, we aimed to explore the bio and chemodiversity of microalgae present in lagoons of New Caledonia, a well-known hotspot of biodiversity [37,38]. Specific environmental conditions (i.e., high UV radiation owing to the leaner ozone layer and high metal concentration of natural origin or caused by mining activity) made these lagoons a source of original microalgae strains with unusual phenotypes, and promising molecules. In this context, microalgae strains were isolated from areas of New Caledonia particularly exposed to metal-rich terrigenous inputs, with strong variation and exposure to sun, salinity, and temperature [37]. We hypothesized that microalgae exposed to these stressful environments might have developed adaptive mechanisms using original secondary metabolites with interesting antioxidant properties. We tested this hypothesis by using four different antioxidant assays, 1,1-diphenyl-2-picrylhydrazyl (DPPH), 2,2’-azino-bis (3-éthylbenzothiazoline-6-sulphonique) (ABTS), oxygen radical absorbance capacity (ORAC), and thiobabituric acid reactive substances (TBARS) coupled with pigment analysis by high performance liquid chromatography (HPLC) to (i) screen and assess the global antioxidant capacities and pigment composition of twelve microalgae species grown at two light intensities, and (ii) to investigate the link between carotenoids concentration and antioxidant properties. 

## 2. Results and Discussion

### 2.1. Antioxidant Activity

To investigate antioxidant activity of microalgae extracts and to consider the complexity of antioxidant actions, we used four different antioxidant assays with different reaction mechanisms. 

DPPH assay measures the ability of a product to quench DPPH radical by electron donation [39]. DPPH quenching capacity of microalgae extract was measured and compared to pure reference compounds of different structural classes. The nature of the molecules tested strongly influences DPPH radical scavenging capacity (Table 1). The best inhibition concentration 50 (IC50) values are observed for trolox (water-soluble α-tocopherol analogue), α-tocopherol, and ascorbic acid (respectively 4.71, 6.20, and 8.73 µg·mL^−1^). The capacity of carotenoids (astaxanthin and β-carotene) to scavenge DPPH radical is weaker, on average 50 times lower than trolox (IC50 of 228.59 and 257.33 µg·mL^−1^). These results are consistent with Müller et al. [40] who found no DPPH radical scavenging activity among 19 carotenoids. Microalgae extracts also present low capacity to quench DPPH radical. The best IC50 value obtained for *Nephroselmis* sp. high light (HL) (395.93 µg·mL^−1^) is 84 times higher than trolox. Furthermore, nine extracts were found to be inactive (IC50 > 1000 µg·mL^−1^ for *Tetraselmis* sp. HL, *Picochlorum* sp. low light (LL), *Schyzochlamydella* sp. LL and HL, *Nitzschia* sp. A HL, *Nitzschia* sp. B LL and HL, *Thalassiosira weissflogi* HL, and *Entomoneis punctulata* LL). ABTS assay measures the capacity of a product to scavenge ABTS radical cation by either direct reduction via electron donation or by hydrogen atom transfer [39]. Results of ABTS assay follow the same trends as results of DPPH assay with some exceptions (Table 1). The best IC50 values are also obtained with ascorbic acid, trolox, and α-tocopherol (respectively 6.08, 6.36, and 10.78 µg·mL^−1^) but activities of β-carotene and astaxanthin measured with ABTS assay are better than with DPPH assay activities. Equally, microalgae extracts are on average 1.5 times more active toward ABTS radical cation than DPPH radical. However, activities of microalgae extracts measured with ABTS assay are still low compared to reference compounds, with IC50 32 (*Tetraselmis* sp. LL) to 161 (*Picochlorum* sp. LL) times higher than ascorbic acid when activities were sufficient to be measured. 

ORAC assay measures the scavenging capacity of a product against peroxyl radicals by hydrogen atom transfer. Trolox is used as reference and results are expressed in trolox equivalent (TE). Microalgae extracts are much more efficient to scavenge peroxyl radicals than DPPH and ABTS radicals. The best antioxidant activities measured with ORAC assays (Table 1) were obtained for *Chaetoceros* sp. HL (190.30 µg TE·mg^−1^) and *Nephroslemis* sp. HL (188.32 µg TE·mg^−1^), with only a factor of five difference compared to trolox. The lowest activities are measured for *Thalassiosira weissflogi* HL (27.71 µg TE·mg^−1^) and *Schizochlamydella* sp. LL and HL (no activity measured) as for DPPH and ABTS assays. 

TBARS assay measures the capacity of a product to inhibit the chain reaction of lipid peroxidation initiated by the ferrous-ascorbate system. Antioxidant can stop the chain reaction by scavenging free radicals but also by limiting the formation of the radicals by metal chelation [41]. The best IC50 are obtained with reference compounds trolox (0.24 µg·mL^−1^) and α-tocopherol (1.30 µg·mL^−1^). Conversely no inhibition of lipid peroxidation was observed with β-carotene and astaxanthin (Table 1). Extracts of *Tetraselmis* sp. at both light intensity (15.43 and 22.77 µg·mL^−1^ for LL and HL, respectively), *Nitzschia* sp. A LL (24.63 µg·mL^−1^), and *Nephroselmis* sp. HL (31.40 µg·mL^−1^) are the most active extracts against lipid peroxidation whereas *Entomoneis punctulata* HL (473.56 µg·mL^−1^) and *Nitzschia* sp. B LL and HL (190.91 and 202.28 µg·mL^−1^) are the less active. 

As expected, inter- and intra-microalgae classes variations were observed for antioxidant activities. Microalgae of the same genus could even have very different antioxidant activity. For example, *Nitzschia* sp. A, especially in LL, can prevent lipid peroxidation and scavenge peroxyl radical, whereas *Nitzschia* sp. B is inactive. It was already noticed by other authors [10,13,42] who found strong variations of radical scavenging capacity of different species of *Chlorella*, *Porphyridium*, or *Nannochloropsis* and even with different strains of a given species.

According to the assay used, the results showed large variations of antioxidant activity from microalgae extracts. For example, *Tetraselmis* sp. extracts are the most active to prevent lipid peroxidation in TBARS assay whereas they have low antioxidant action toward DPPH radical and peroxyl radical in ORAC assay. Similarly, *Chaetoceros* sp. HL is the most efficient extract against peroxyl radical whereas it has almost no effect on scavenging DPPH and ABTS radicals and to inhibit lipid peroxidation. Those different antioxidant activities of microalgae extracts in specific tests confirm the need to use several assays with different mechanisms of action to evaluate antioxidant capacities of natural extracts as supported by other authors [39,43,44,45]. 

The results obtained with the four assays reveal that none of the microalgae studied has an interesting activity against DPPH and ABTS radicals compared to reference compounds. The best results to scavenge peroxyl radical was achieved by *Chaetoceros* sp., *Nephroselmis* sp., and *Nitzschia* sp. A. The last two species also have the capacity to prevent lipid peroxidation as much as *Tetraselmis* sp. In published data about evaluation of microalgae as natural antioxidant, assays used differ in method (i.e., extraction procedure, solvent, substrate, time of reaction, and concentration), data units, and analysis. Furthermore, in most assays, no comparison to reference compounds is made that hampers comparison between studies and highlight the need to standardized procedures used in antioxidant studies.

### 2.2. Carotenoids

**Figure 1:**
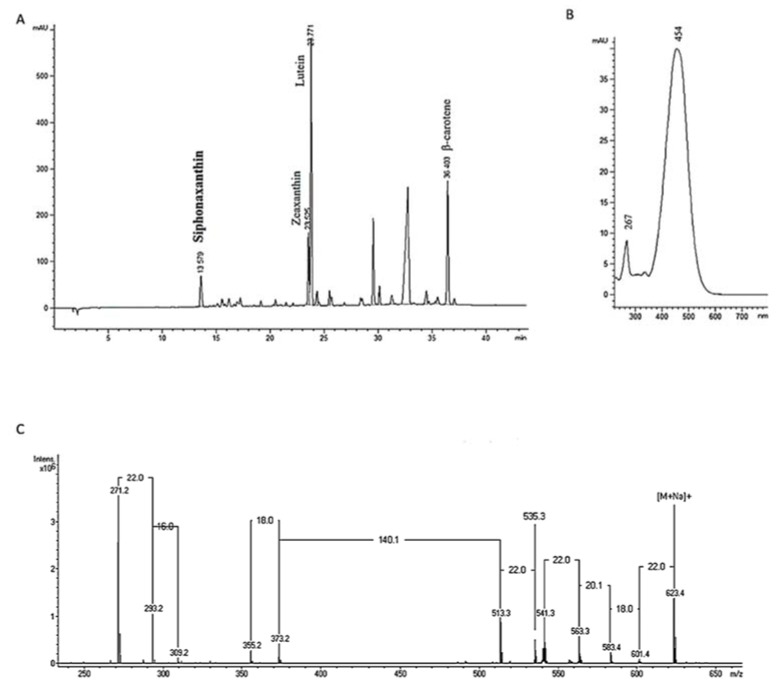
Identification and characterization of siphonaxanthin: HPLC chromatogram at 450 nm of *Nephroselmis* sp. HL crude extract (**A**), UV-vis spectrum in HPLC system (**B**), and mass spectrum of siphonaxanthin (**C**).

To investigate the link between carotenoid content and antioxidant activity of microalgae, the carotenoid content of microalgae MeOH/DCM extracts was determined by HPLC and UV/Visible detection. The carotenoid analysis of microalgae extracts reveals large variations of carotenoid concentration and composition (Table 2). *Nephroselmis* sp. HL has the higher concentration of total carotenoid (66.89 µg·mg^−1^), 1.7 times more than *Nitzschia* sp. A HL (38.20 µg·mg^−1^), which has the second highest content, followed by *Nitzschia* sp. LL (28.80 µg·mg^−1^). *Thalassiosira weissflogi* HL (0.10 µg·mg^−1^) and *Schizochlamydella* sp. HL (0.18 µg·mg^−1^) and LL (2.29 µg·mg^−1^) showed the lowest content of total carotenoids. With the exception of β-carotene that is common to all species, we can distinguish two groups from carotenoids composition corresponding, classically, to the phyla of Chlorophyta and Bacillariophyta (Figure S1) [46]. In species belonging to Chlorophyta (*Nephroselmis* sp., *Tetraselmis* sp., *Dunaliella* sp., *Picochlorum* sp., and *Schizochlamydella* sp.) lutein and zeaxanthin in addition to β-carotene are the major carotenoids. With the exception of *Nephroselmis* sp., lutein represents more than 50% of total carotenoids, followed by 9% to 31% of β-carotene and 8% to 23% of zeaxanthin. *Nephroselmis* sp., compared to other Chlorophyte species, is characterized by a higher level of zeaxanthin which represents more than 50% of total carotenoids for both light conditions. This species also has the highest level of β-carotene for both light intensities, the highest content in lutein in HL condition, and an interesting pigment with UV-vis spectrum and mass spectrometry similar to siphonaxanthin (Figure 1) [47]. This xanthophyll is mainly found in Ulvophyceae, Chlorophyceae, and Prasinophyceae and has already been described in *Nephroselmis* genus [47]. It exhibits antioxidant activity [48] but also anti-angiogenic effect [49], apoptosis-inducing effects [50], and can inhibit adipogenesis [51]. In species belonging to Bacillariophyta (*Nitzschia* sp. A and B, *Thalassiosira weissflogi*, *Entomoneis punctulata*, *Cylindrotheca closterium*, *Chaetoceros* sp., and *Bacillaria* sp.), fucoxanthin is the major carotenoid, representing more than 70% of total carotenoids in all species. The highest concentration of this carotenoid is measured in *Nitzschia* sp. A in both light conditions (32.30 µg·mg^−1^ HL and 22.40 µg·mg^−1^ LL). Bacillariophytes are also characterized by the presence of cis-fucoxanthin (4% to 16% of total carotenoids), diatoxanthin (1% to 15% of total carotenoids), and smaller amounts of β-carotene than Chlorophytes (2% to 9% of total carotenoids). 

Light intensity strongly influences carotenoid content and composition, and its effects seems species specific. Indeed, *Nephroselmis* sp., *Dunaliella* sp., *Picochlorum* sp., *Nitzschia* sp. A, and *Entomoneis punctulata*, has higher total carotenoid and individual carotenoids content with HL intensity, whereas the opposite is observed for *Tetraselmis* sp., *Schizochlamydella* sp., *Nitzschia* sp. B, *Thalassiosira weissflogi*, *Cylindrotheca closterium,* and *Chaetoceros* sp. (Table 2). Carotenoids are usually separated in two categories: Primary carotenoids located in the photosynthetic apparatus, that act as accessory light harvesting pigment or with protective function, and secondary carotenoids separated from photosynthetic apparatus that have mainly photoprotective functions. When microalgae are exposed to light-excess conditions, photosynthetic pigments (chlorophyll and primary carotenoids) generally decrease whereas secondary carotenoids increase in some chlorophytes species [52,53]. It could explain the different effect of light intensity on carotenoid content observed in this study. For species belonging to Bacillariophyta, carotenoid content is mainly constituted of fucoxanthin, a photosynthetic pigment. As expected there is higher fucoxanthin in LL condition in most species which is in agreement with the litterature [35,54,55]. In Chlorophyte species, lutein is the major carotenoid. It is a primary carotenoid with both accessory light harvesting and photoprotective functions [53]. As a primary pigment, we expected that lutein content decrease with increasing light intensity as in *Tetraselmis* sp and *Schizochlamydella* sp. However, there is higher lutein content in HL condition for *Nephroselmis* sp., *Dunaliella* sp., and *Picochlorum* sp. Contrasted results are also observed in the literature according to species: Lutein accumulation was observed with increasing light intensity in *Parachlorella* sp. [56] whereas a decreased was measured in *Desmodesmus* sp., *Muriellopsis* sp., and *Chlorella zofingiensis* [57,58,59].

**Figure 2:**
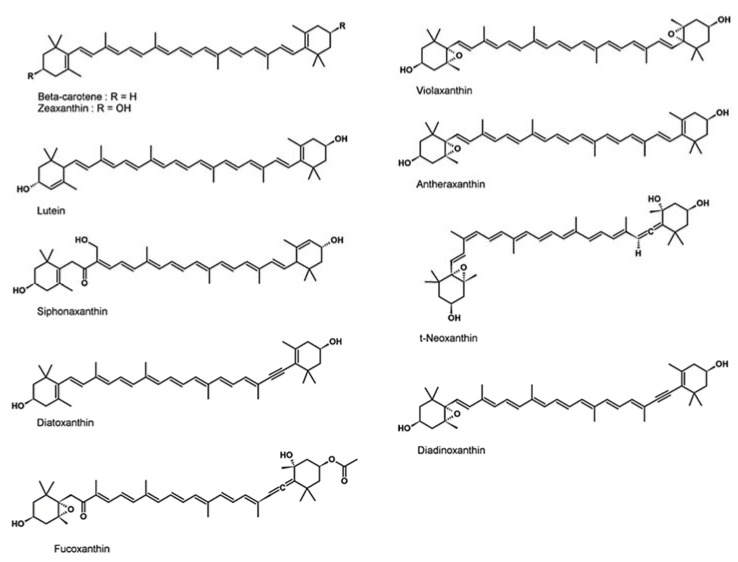
Carotenoids structure.

Another extracting method was performed using 95% aqueous acetone. In these extracts, the distribution pattern of the carotenoids is different compared to MeOH/DCM extracts. Acetone fresh extracts are characterized by the presence, besides carotenoids detected in MeOH/DCM extracts, of diadinoxanthin, violaxanthin, antheraxanthin, and a significant increase in t-neoxanthin concentration while minor changes are observed for other carotenoids (Table 3). All absent compounds in MeOH/DCM extracts belong to the subclass of xanthophyll 5,6-epoxides (Figure 2) which are known to be sensible to degradations by heat through epoxide isomerization [60]. The internal constraint of 5,6-epoxy ring causes a subsequent rearrangement to a 5,8-dihydrofuran ring that give compounds which are then degraded by the oxidation process. This mechanism of action is further highlighted in our experiments by partial or non-degradation of fucoxanthin in MeOH/DCM extracts which is the only xanthophyll 5,6-epoxide to have its position eight occupied by a ketone group that blocks rearrangement to a 5,8-dihydrofuran ring. In this case, the epoxide isomerization results in a partial isomerization of fucoxanthin into cis-fucoxanthin which is not observed when carotenoids analyses are performed on fresh acetone extracts [61,62].

### 2.3. Correlation between Antioxidant Activity and Carotenoid Content

With the aim to highlight a link between antioxidant activity and carotenoid content of the microalgae extract, a correlation analysis was performed (Table 4). However, since no interesting antioxidant activities were measured with DPPH and ABTS, the results of these assays were not considered. 

The correlation analysis reveals a strong positive correlation (correlation coefficient of 0.71) between antioxidant activity measured with ORAC assay and total carotenoid content. However, the R² value (0.51) suggests that besides carotenoids, other compounds contributed to the antioxidant activity measured in the microalgae extracts. A closer look to carotenoid composition indicates that xanthophylls contribute greatly (correlation coefficient of 0.71) to the correlation with antioxidant activity measured with ORAC assay, specifically lutein for species belonging to Chlorophytes (correlation coefficient of 0.78, R² of 0.60). On the other hand, β-carotene content is not correlated with the antioxidant activity measured with ORAC assay. 

Considering TBARS assay, correlation analysis shows that carotenoids do not contribute to the antioxidant activity measured (correlation coefficients non-significant). Others types of molecules are involved to prevent lipid peroxidation. This inhibition might be explained by phenolic [63,64] and fatty acid compounds present in the extracts. However, phenolic compounds are probably not the molecules involved in our study as no activities is found using DPPH and ABTS assays, whereas these assays are known to highlight antioxidant activity of polyphenols [65,66]. Since the solvent mixture, MeOH/DCM, is commonly used for lipid extraction [67], a significant amount of lipids could be present in our extracts and could explain the results on antioxidant activities. Indeed, Custodio et al. [68] showed that *Tetraselmis chuii*, *Nannochloropsis oculata*, *Chlorella minutissima,* and *Rhodomonas salina* have radical scavenging and metal chelating activity, and hypothesized that it is related to the high abundance of polyunsaturated fatty acid (PUFA) in their algal extracts. Yoshida et al. [69] also demonstrated that phosphatidylcholine, a phospholipid, can inhibit lipid peroxidation induced by Fe-ascorbate system by chelating iron. 

### 2.4. Effect of Light Intensity on Antioxidant Activity

Microalgae were cultivated at two light intensities (250 at LL to 600 µmol·m^−2^·s^−1^ at HL) to evaluate the impact of this key factor on antioxidant activity. The light intensity applied to microalgae culture has an influence on anti-radical activity measured with DPPH and ABTS assays (Table 1). However, these activities remain well below activities measured with trolox, α-tocopherol, and ascorbic acid regardless light intensity. Light intensity has a strong effect on antioxidant activity measured with ORAC assay (*p* < 0.001), e.g., *Dunaliella* sp. antioxidant activity was doubled by increasing light intensity. However, according to species, light intensity can have contrasting effects on antioxidant activity measured with ORAC assay. For *Nephroselmis* sp., *Dunaliella* sp., *Picochlorum* sp., *Nitzschia* sp. B, *Entomoneis punctulata*, *Cylindrotheca closterium,* and *Chaetoceros* sp., increasing light intensity from 250 to 600 µmol·m^−2^·s^−1^ led to an increase of the antioxidant activity contrary to *Tetraselmis* sp., *Nitzschia* sp. A, and *Thalassiosira weissflogi*. 

Light intensity influences positively or negatively the capacity of microalgae extracts (except *Nitzschia* sp. B) to inhibit lipid peroxidation with TBARS assay. Antioxidant activity measured with TBARS assay is maximized with LL intensity for most microalgae species in contrast to results observed with ORAC assay. Indeed, apart from *Nephroselmis* sp. and *Schizochlamydella* sp., increasing light intensity causes a decrease of the antioxidant capacity of all species up to four folds (e.g., *Nitzschia* sp. A). We hypothesized that antioxidant activity measured with TBARS assay could be related to PUFA content. In that case, higher PUFA levels would be measured in LL culture condition. It is consistent with numerous studies that suggest that PUFA content is inversely related to growth light intensity in most microalgae species [70,71,72,73,74,75].

The contrasted effects of light intensity on results highlight that the assays used are more or less specific to given antioxidant molecules present in the extracts. Overall, high light intensity promotes the production of compounds able to scavenge peroxyl radical, whereas low light intensity promotes compounds that inhibit lipid peroxidation. It implies that light intensity will drive the antioxidant production towards one type of molecules instead of the other. However, *Nephroselmis* sp. and *Nitzschia* sp. A both have the capacity to limit lipid peroxidation and to scavenge peroxyl radicals in HL conditions and LL conditions, respectively. Those contrasted results highlight the need for further photophysiological investigations to link antioxidant capacity to light history and biochemical composition of microalgae species. 

Few studies explored the impact of light intensity on the global antioxidant activity of microalgae. Published results focus on the effects of culture conditions on specific antioxidant compounds, especially carotenoids. Nevertheless, some studies revealed significant effect of light intensity on antioxidant molecules and highlight that this result is often species-specific. For example, Zhang et al. [76] showed that increasing light intensity from 40 to 200 µmol·m^−2^·s^−1^ led to a decrease of β-carotene and superoxide dismustase in *Chaetoceros calcitrans* whereas it led to an increase of both molecules in *Thalassiosira weissflogi* and high light combined with other abiotic stresses stimulates the synthesis of astaxanthin and β-carotene in *Haematococcus pluvialis* [77,78,79] and *Dunaliella salina* [31,32,33], respectively.

## 3. Materials and Methods 

### 3.1. Strains

Twelve species of microalgae isolated in New Caledonia have been selected for their ease of handling and high growth potential [37]. Authorizations for the sampling were delivered by the South Province of New Caledonia (n°26960, n°1546, and n°9705) and the North Province of New Caledonia (n°609011-55 and n°609011-54). The 12 species belong to six classes: Five of them are Bacillariophyceae; *Cylindrotheca closterium*, *Nitzschia* sp. A, *Nitzschia* sp. B, *Bacillaria* sp., and *Entomoneis punctulata*, two strains belong to Mediophyceae; *Chaetoceros* sp. and *Thalassiosira weissflogi,* two of them are Trebouxiophyceae; *Picochlorum* sp. and *Schizochlamydella* sp., one strain belongs to Chlorophyceae; *Dunaliella* sp., one strain is a Chlorodendrophyceae; *Tetraselmis* sp., and the last strain *Nephroselmis* sp. belongs to Nephrophyceae. 

### 3.2. Culture Conditions

For antioxidant assays, microalgae were cultivated in 10 L air bubbled balloon in batch condition. They were inoculated by seven day old cultures grown in the same conditions. Cultures were done in Conway-enriched seawater [80] filtered at 0.2 µm and sterilized. Temperature was set at 28 °C ± 1, and pH regulated at 7.5 ± 0.3 by CO_2_ injection. Continuous light was applied and set using a Li-cor quantum meter (LI-250A) with a spherical probe (US-SQS/L) at two different intensities of 250 µmol·m^−2^·s^−1^ (low light condition) and 600 µmol·m-^2^·s^−1^ (high light condition) to all species, except for *Bacillaria* sp. which was unable to grow in HL condition. At stationary growth phase, microalgae biomasses were harvested by centrifugation, freeze dried, and kept at −20 °C until extraction.

For pigment analysis, microalgae were grown in sterile 1 L flasks in the same conditions as 10 L cultures for antioxidant assays without pH regulation. Cells were harvested by centrifugation, freeze dried, and kept at −80 °C until analysis.

### 3.3. Extraction

Two extraction protocols were applied, a first one for antioxidant assays and pigments quantification with a mixture of solvent with a broad polarity to extract a large variety of secondary metabolite, and a second one for the specific characterization of the microalgae pigment composition. Methanol/dichloromethane (MeOH/DCM) dried extracts for evaluation of antioxidant activity were obtained by suspending freeze dried biomasses (1.5 to 5.5 g) from 10 L cultures in 100 mL of MeOH/DCM mixture (50:50 v/v), and submitted to ultrasound for 60 min. Extracts were then filtered and the process was repeated until the biomass became colorless. The crude MEOH/DCM extracts were pooled and dried under vacuum in a rotary evaporator at 30 °C. 

Fresh acetone extracts for pigments analysis were obtained by suspending 1 mg of freeze dried biomass from 1 L cultures in 1 mL of acetone 95% and submitted to ultrasound 10 min in an ice bath.

### 3.4. DPPH Assay

DPPH assay measures the capacity of an antioxidant to scavenge DPPH radical by electron donation. In presence of radical scavenger, purple DPPH radical is reduced to a pale yellow compound and the discoloration of the radical is measured at 515 nm [39]. DPPH radical scavenging capacity of microalgae extracts was evaluated with the slightly modified method of Kenny et al. [81]. Trolox, ascorbic acid, α-tocopherol, β-carotene, and astaxanthin were used as reference compounds. MeOH/DCM dried extracts were diluted in ethanol at a concentration ranging from 20 to 1000 µg·mL^−1^ and loaded (100 µL) in 96 well plates. The same volume of reference compounds (0.5–500 µg·mL^−1^) and ethanol (blank) were placed in the wells. Then, 100 µL of DPPH (0.12 M in ethanol) was added. To prevent interference from carotenoids, a control was performed by adding 100 µL of ethanol instead of DPPH. After an incubation of 30 min in darkness at room temperature, absorbance at 515 nm was measured. Percentage of inhibition of DPPH (I%) was calculated for each sample with the following equation:

(1) $$I\%=\lfloor \frac{\left(A_{blank}-A_{sample}\right)}{A_{blank}}\rfloor \times 100$$

where *A_blank_* is the absorbance at 515 nm of DPPH in ethanol and *A_sample_* is the absorbance at 515 nm of the sample minus the absorbance of the control with ethanol instead of DPPH.

The results are expressed as IC50, the concentration needed to scavenge 50% of radical. It was determined by linear regression by plotting concentration of each extract or reference compound with their corresponding I%. 

### 3.5. ABTS Assay

In ABTS assay, antioxidants scavenge the blue chromophore ABTS radical cation (ABTS^•+^) by either electron donation or hydrogen electron transfer [39]. It induces a discoloration that can be followed at 734 nm. ABTS assay was applied to microalgae MeOH/DCM dried extracts according to Re et al. [82] with modifications to take colored algal material into account and to fit with 96 well plates. Reference compounds were similar to the one used in the DPPH assay. ABTS^+^ was generated by mixing 2.45 mM of potassium persulfate and 7 mM ABTS solution and placed 12 to 16 h in darkness at room temperature before use. Microalgae MeOH/DCM dried extracts and reference compounds were diluted in ethanol at a concentration ranging from 10 to 1000 µg·mL^−1^ and 0.5 to 500 µg·mL^−1^, respectively. Then 100 µL of each sample were placed in 96 well plates. ABTS^+^ solution was diluted with ethanol to have an absorbance of 0.70 ± 0.02, and 100 µL of the mixture was added to the wells. Controls containing ethanol instead of ABTS^+^ were performed to prevent pigment interferences. Immediately after 6 min of incubation at 30°C, the absorbance was measured at 734 nm. Percentage of inhibition was calculated with the same equation than for DPPH (Equation (1)) where *A_blank_* is the absorbance at 734 nm of ABTS^+^ in ethanol, and *A_sample_* the absorbance at 734 nm of the sample minus the absorbance of the control with ethanol instead of ABTS^+^. For each microalgae extract and reference compounds, IC50 values were calculated as described before.

### 3.6. ORAC Assay

ORAC assay measures the chain breaking capacity of an antioxidant against peroxyl radicals by hydrogen atom transfer. Peroxyl radicals, induced by the thermal decomposition of 2,2′-azobis-(2-amidinopropane) dihydrochloride (AAPH), react with a fluorescent probe (fluorescein), causing a fluorescence loss over time that is measured [39]. According to Watanabe et al. [83], microalgae MeOH/DCM dried extracts were diluted (3.125 to 100 µg·mL^−1^) in mixture containing DMSO/diluent 10:90 (v/v) with diluent made up of 7% (w/v) of randomly methylated β-cyclodextrin (RMCD) in 50% (v/v) acetone aqueous solution. Trolox (0.5 to 10 µg·mL^−1^), used as standard, was diluted in the same mixture DMSO/diluent. Each sample was loaded (35 µL) in 96 wells plate, and the same volume of DMSO/diluent was used as blank. Then 115 µL of fluorescein (77.5 nM) was added to the wells. After 10 min of incubation at 37 °C with agitation at 20 rpm, 50 µL of AAPH (82.4 mM) was added. Fluorescence decay was measured every 2 min for 300 min at an excitation wavelength of 485 nm and emission wavelength of 528 nm. 

Area under the curve (AUC) for each sample was calculated with the following formula from Huang et al. [84]:

(2) $$\mathrm{AUC}=0.5+\frac{f_{1}}{f_{0}}+\ldots \frac{f_{i}}{f_{0}}+\ldots +\frac{f_{298}}{f_{0}}+0.5\left(\frac{f_{300}}{f_{0}}\right)$$

where *f_o_* is the initial fluorescence and *f_i_* is the fluorescence at time *i*. Net AUC was obtained by subtracting AUC of the blank to the sample. The calibration curve of trolox was constructed by plotting trolox concentration versus net AUC and used for the quantification of antioxidant activity of the microalgae extracts by linear regression. The results are expressed as ORAC value in µg trolox equivalent·mg^−1^ of extract.

### 3.7. TBARS Assay

TBARS assay measures antioxidant capacity to inhibit lipid peroxidation. The degradation of lipids leads to the formation of malondialdehyde (MDA) that reacts with thiobarbituric acid (TBA) to form a red complex that can be followed at 534 nm [85]. The method of Ahmed et al. [86] was applied on microalgae MeOH/ extract with some modifications to fit with algal material. Fe-ascorbate system was chosen for oxidation catalysis with linoleic acid as the source of unsaturated fatty acid. Linoleic acid (0.2 mL) was emulsified with Tween 20 (0.4 mL) and phosphate buffer (19.4 mL, 20 mM, pH 7.4). Microalgae MeOH/DCM dried extracts (0.5 mL,15.625 to 500 µg·mL^−1^) or reference compounds (0,5 mL, 0.03 to 5 µg·mL^−1^) diluted in ethanol were mixed with phosphate buffer (0.6 mL), FeSO_4_ (0.2 mL, 0.01%), ascorbic acid (0.2 mL, 0.01%), and linoleic emulsion (0.5 mL). Ascorbic acid, as part of the catalysis system, has not been tested as a reference compound. Blank samples were made by substituting microalgae extract with the same volume of ethanol. After 24 h of incubation at 37 °C, oxidation was stopped by mixing 0.4 mL of each sample with butylated hydroxytoluene (BHT) (0.04 mL, 0.4%). Then a mixture (0.44 mL) of TBA (0.8%) and trichloroacetic acid (TCA) (4%) was added. To prevent pigment interferences, controls containing phosphate buffer instead of TBA/TCA were performed. The samples were incubated at 100°C for 30 min, and then cooled and centrifuged. The absorbance of the supernatant was measured at 534 nm. The percentage of inhibition of linoleic acid peroxidation was calculated with (Equation (1)) as for DPPH and ABTS assay where *A_blank_* is the absorbance at 534 nm of blank sample with ethanol, and *A_sample_* is the absorbance at 534 nm of the sample minus the absorbance of the control with phosphate buffer instead of TBA/TCA. For each microalgae extract and reference compounds, IC50 values was calculated from regression lines by plotting percentage of inhibition of linoleic acid peroxidation with their corresponding extracts concentrations.

### 3.8. Pigments Analysis

Pigments analysis was performed on fresh 95% aqueous acetone extracts to characterize lipophilic pigment composition and on MeOH/DCM dried extracts to study the relationship between carotenoids content and antioxidant activity.

Just after extraction, fresh acetone extracts were filtered on a 0.2 µM PTFE filter before immediate HPLC analysis. MeOH/DCM dried extracts were solubilized in ethanol at 0.5 mg mL^−1^ and filtered on a 0.2 µM PTFE filter before HPLC analysis. The samples were analyzed by HPLC-UV-DAD (Agilent Technologies, Santa Clara, CA, United States, series 1200 HPLC-UV-DAD) using an Eclipse XDB-C8 reverse phase column (150 by 4.6 mm, 3.5 µm particle size, Agilent Technologies) following the method by Van Heukelem and Thomas [87]. HPLC grade MeOH and water were purchased from Merck Chemicals (Darmstadt, Germany) and tetrabutyl ammonium acetate from Sigma-Aldrich (Darmstadt, Germany). Quantification was carried out using external calibration against pigments standard (lutein, neoxanthin, violaxanthin, antheraxanthin, zeaxanthin, β-carotene, diatoxanthin, diadinoxantin, and fucoxanthin provided by DHI, Denmark). Quantification of siphonaxanthin was done according to fucoxanthin standard as recommended by Roy et al. [88].

### 3.9. Mass Spectrometry Analysis of Siphonaxanthin

One unidentified pigment in *Nephroselmis* sp. was also analyzed by mass spectrometry (MS) analysis using an ion trap Bruker Esquire HCT Ultra MS instrument equipped with an electrospray ion source in positive mode (data were viewed by using Hystar Bruker software). HPLC quality solvents were purchased from Fischer Chemicals (Leicestershire, UK). Dried extract of *Nephroselmis* sp. HL was dissolved in methanol/acetone 50:50 (v/v) at 5 mg·mL^−1^. Optimized pseudo isocratic elution was applied on a RP C18ec Macherey Nagel Nucleodur C18ec (4.6 by 250 mm) column and using as solvent A, water plus formic acid 0.05%, and as solvent B (methanol plus formic acid 0.05%). The analytical conditions were as follows: Flow rate one mLPmin^−1^, injection volume of 50 µL, 10 min 5% of A to 0% of A, then 35 min 100% of B.

Siphonaxanthin characterization (Figure 1): UV/VIS (ethanol) λmax (retention time): 267, 454 nm (6.7 min), MS-ESI + *m*/*z*: 623.4 [M + Na]^+^; 601.4 [M + H]^+^; and 583.4 [M + H-H_2_O]^+^. 

### 3.10. Statistical Analysis

Data in tables and text are expressed as mean ± standard deviation (SD). Normality and equality of variance were tested and depending on the results, statistical analyses consisted of analysis of variance (ANOVA) or Kruskal–Wallis test followed by a Tuckey Test or a Mann–Whitney test. Significant effects of light, species, and the interaction of the two factors on antioxidant activity were tested with a two-way ANOVA when possible. Pearson correlation test was used to study the relationship between antioxidant activity and carotenoids content. Differences were considered significant at *p <* 0.05. All tests were performed with Statgraphics Centurion XV.I (StatPoint Technologies, Inc., Warrenton, VA, United States).

## 4. Conclusions

The results of the four antioxidant assays highlight the need to use several assays with different modes of action to investigate the most comprehensive antioxidant activity of natural extracts. Indeed, none of the twelve microalgae tested have the capacity to scavenge DPPH and ABTS radicals but they can scavenge peroxyl radical (*Chaetoceros* sp., *Nephroselmis* sp., and *Nitzschia A* sp.) and inhibit lipid peroxidation (*Tetraselmis* sp., *Nitzschia* A sp., and *Nephroselmis* sp.). These antioxidant properties are linked to the biochemical composition of the microalgae: Peroxyl radical scavenging capacity measured with ORAC assay is correlated to xanthophylls whereas lipid peroxidation inhibition measured with TBARS assay is related to other compounds that may be PUFA. 

The carotenoid detected on fresh acetone extracts and MeOH/DCM extracts showed different profiles according to extraction methods and owing to the thermal degradation of the xanthophyll 5,6-epoxides (violaxanthin, diadinoxanthin, and antheraxanthin). Otherwise, carotenoid composition of microalgae extracts is typical of the studied species, but highlights the possibility to produce pigment of interests, such as siphonaxanthin, with microalgae. The siphonaxanthin has several bioactive properties, including antioxidant activity; nonetheless, the effects of culture conditions on its production by microalgae have not yet been investigated. The present results showed that light intensity is a key factor to influence global antioxidant activity of microalgae. Indeed, for most species tested, HL intensity increases peroxyl radical scavenging capacity whereas LL intensity increases lipid peroxidation inhibition. Other parameters (temperature, pH, salinity, nutrient, etc.) are known to impact biochemical content of microalgae. Thus, it would be interesting to study the effects of these parameters to optimize antioxidant production, especially siphonaxanthin production by *Nephroselmis* sp. 

## Figures and Tables

**Table 1:** Antioxidant activities of reference compounds and microalgae extracts cultivated at two light intensities, 250 µmol·m^−2^·s^−1^ (low light (LL)) and 600 µmol·m^−2^·s^−1^ (high light (HL)). Different letters in the same column indicate a statically significant difference (*p* < 0.05).

		DPPH	ABTS	ORAC	TBARS
		(IC_50_ in µg of dry extract·mL^−1^)	(IC_50_ in µg of dry extract·mL^−1^)	(µg Trolox equivalent·mg^−1^ of dry extract)	(IC_50_ in µg of dry extract·mL^−1^)
*Nephroselmis* sp.	LL	695.80 ± 57.28 ^hi^	558.16 ± 70.02 ^j^	138.82 ± 0.88 ^f^	63.39 ± 5.04 ^h^
	HL	395.93 ± 70.98 ^f^	311.08 ± 26.80 ^f^	188.32 ± 0.51 ^b^	31.40 ± 2.13 ^e^
*Tetraselmis* sp.	LL	753.99 ± 81.35 ^jk^	193.17 ± 11.18 ^e^	110.48 ± 0.71 ^i^	15.43 ± 2.47 ^c^
	HL	>1000	341.38 ± 28.86 ^g^	89.16 ± 1.51 ^o^	22.77 ± 4.54 ^d^
*Dunaliella* sp.	LL	823.98 ± 77.14 ^kl^	430.69 ± 31.48 ^h^	59.51 ± 1.47 ^s^	58.20 ± 8.35 ^gh^
	HL	892.18 ± 67.60 ^m^	794.54 ± 64.60 ^m^	141.53 ± 0.79 ^e^	68.24 ± 5.65 ^i^
*Picochlorum* sp.	LL	>1000	981.96 ± 40.66 ^o^	55.17 ± 0.68 ^t^	42.10 ± 5.87 ^f^
	HL	671.50 ± 61.75 ^h^	463.90 ± 17.30 ^i^	98.64 ± 0.80 ^l^	87.76 ± 8.36 ^k^
*Schizochlamydella* sp.	LL	>1000	>1000	n.d.	55.41 ± 3.72 ^g^
	HL	>1000	>1000	n.d.	43.51 ± 8.88 ^f^
*Nitzschia* sp. *A*	LL	497.27 ± 79.37 ^g^	462.96 ± 17.88 ^i^	179.75 ± 0.78 ^c^	24.63 ± 6.07 ^d^
	HL	>1000	>1000	119.76 ± 1.49 ^h^	98.77 ± 7.73 ^l^
*Nitzschia* sp. *B*	LL	>1000	>1000	78.95 ± 1.54 ^p^	190.91 ± 24.36 ^p^
	HL	>1000	>1000	92.02 ± 1.52 ^n^	202.28 ± 27.86 ^p^
*Thalassiosira weissflogi*	LL	939.31 ± 104.41 ^n^	620.26 ± 54.67 ^k^	69.99 ± 1.49 ^q^	114.58 ± 6.69 ^m^
	HL	>1000	>1000	27.71 ± 0.95 ^u^	164.44 ± 5.35 ^o^
*Entomoneis punctulata*	LL	>1000	>1000	68.09 ± 1.58 ^r^	147.34 ± 17.47 ^n^
	HL	839.30 ± 84.45 ^lm^	>1000	94.20 ± 1.45 ^m^	473.56 ± 66.26 ^q^
*Cylindrotheca closterium*	LL	890.75 ± 72.49 ^mn^	615.65 ± 27.05 ^k^	105.48 ± 1.58 ^j^	79.67 ± 11.87 ^j^
	HL	710.60 ± 61.83 ^hij^	654.79 ± 21.27 ^l^	127.14 ± 1.29 ^g^	103.48 ± 15.18 ^l^
*Chaetoceros* sp.	LL	484.47 ± 87.98 ^g^	441.03 ± 17.20 ^h^	170.00 ± 0.57 ^d^	77.97 ± 6.16 ^j^
	HL	773.52 ± 68.35 ^k^	791.40 ± 49.81 ^m^	190.3 ± 0.78 ^a^	116.08 ± 17.32 ^m^
*Bacillaria* sp.	LL	749.55 ± 87.70 ^ij^	895.81 ± 44.93 ^n^	102.19 ± 1.45 ^k^	60.14 ± 8.54 ^gh^
Trolox		4.71 ± 0.53 ^a^	6.36 ± 1.33 ^a^	-	0.24 ± 0.06 ^a^
α-Tocopherol		6.20 ± 0.33 ^b^	10.78 ± 0.26 ^b^	-	1.30 ± 0.16 ^b^
Ascorbic acid		8.73 ± 1.63 ^c^	6.08 ± 0.75 ^a^	-	-
β-Carotene		257.33 ± 20.89 ^e^	37.04 ± 2.56 ^c^	-	>200
Astaxanthin		228.59 ± 41.71 ^d^	98.54 ± 6.58 ^d^	-	>200

n.d.: Not detected.

**Table 2:** Quantification of carotenoids (µg.mg^−1^ of extract) in MeOH/DCM dried extracts of microalgae cultivated at two light intensities, 250 µmol·m^−2^·s^−1^ (LL) and 600 µmol·m^−2^·s^−1^ (HL). Lut, lutein; t-Neo, t-neoxanthin; Siph, siphonaxanthin; Zea, zeaxanthin; β-Car, β-carotene; Fuco, fucoxanthin; cis-Fuco, cis-fucoxanthin; and Dt, diatoxanthin.

			Lut	t-Neo	Siph	Zea	β-Car	Fuco	Cis-Fuco	Dt	Total Carotenoids
Chlorophyta	*Nephroselmis* sp.	LL	4.70	n.d.	4.11	13.60	5.40	n.d.	n.d.	n.d.	27.81
		HL	13.50	n.d.	6.89	39.30	7.20	n.d.	n.d.	n.d.	66.89
	*Tetraselmis* sp.	LL	9.51	1.43	n.d.	1.91	4.42	n.d.	n.d.	n.d.	17.27
		HL	7.04	1.38	n.d.	1.76	3.01	n.d.	n.d.	n.d.	13.19
	*Dunaliella* sp.	LL	3.36	0.29	n.d.	0.53	1.90	n.d.	n.d.	n.d.	6.08
		HL	4.83	0.15	n.d.	1.55	2.00	n.d.	n.d.	n.d.	8.53
	*Picochlorum* sp.	LL	7.07	n.d.	n.d.	2.32	0.89	n.d.	n.d.	n.d.	10.28
		HL	7.27	0.92	n.d.	2.37	2.79	n.d.	n.d.	n.d.	13.35
	*Schizochlamydella* sp.	LL	1.58	n.d.	n.d.	0.18	0.53	n.d.	n.d.	n.d.	2.29
		HL	0.18	n.d.	n.d.	n.d.	n.d.	n.d.	n.d.	n.d.	0.18
Bacillariophyta	*Nitzschia* sp. *A*	LL	n.d.	n.d.	n.d.	n.d.	1.40	22.40	4.50	0.50	28.80
		HL	n.d.	n.d.	n.d.	1.30	0.90	32.30	2.50	1.20	38.20
	*Nitzschia* sp. *B*	LL	n.d.	n.d.	n.d.	n.d.	0.20	10.30	1.10	0.10	11.70
		HL	n.d.	n.d.	n.d.	n.d.	0.20	7.40	0.30	0.20	8.10
	*Thalassiosira weissflogi*	LL	n.d.	n.d.	n.d.	n.d.	1.30	10.76	1.00	1.40	14.46
		HL	n.d.	n.d.	n.d.	n.d.	n.d.	0.10	n.d.	n.d.	0.10
	*Entomoneis punctulata*	LL	n.d.	n.d.	n.d.	n.d.	n.d.	7.00	0.60	n.d.	7.60
		HL	n.d.	n.d.	n.d.	n.d.	1.50	15.30	2.90	0.60	20.30
	*Cylindrotheca closterium*	LL	n.d.	n.d.	n.d.	n.d.	0.50	12.60	1.30	0.70	15.10
		HL	n.d.	n.d.	n.d.	n.d.	0.40	12.10	0.90	1.30	14.70
	*Chaetoceros* sp.	LL	n.d.	n.d.	n.d.	n.d.	1.30	19.30	2.20	4.00	26.80
		HL	n.d.	n.d.	n.d.	n.d.	n.d.	12.40	1.20	2.40	16.00
	*Bacillaria* sp.	LL	n.d.	n.d.	n.d.	n.d.	1.50	16.30	3.40	0.70	21.90

n.d.: Not detected.

**Table 3:** Quantification of carotenoids (µg.mg^−1^ of biomass) in fresh acetone extracts of microalgae cultivated at two light intensities, 250 µmol·m^−2^·s^−1^ (LL) and 600 µmol·m^−2^·s^−1^ (HL). Lut, lutein; t-Neo, t-neoxanthin; Siph, siphonaxanthin; Zea, zeaxanthin; β-Car, β-carotene; Viola, violaxanthin; Anthe, antheraxanthin; Fuco, fucoxanthin; cis-Fuco, cis-fucoxanthin; Dt, diatoxanthin; and Dd, diadinoxanthin.

			Lut	t-Neo	Siph	Zea	β-Car	Viola	Anthe	Fuco	Cis-Fuco	Dt	Dd	Total Carotenoids
Chlorophyta	*Nephroselmis* sp.	LL	0.48	0.21	0.14	0.68	0.65	0.33	0.11	n.d.	n.d.	n.d.	n.d.	2.60
		HL	0.31	0.11	0.05	0.73	0.36	0.14	0.09	n.d.	n.d.	n.d.	n.d.	1.79
	*Tetraselmis* sp.	LL	1.02	0.39	n.d.	0.59	0.92	0.47	0.08	n.d.	n.d.	n.d.	n.d.	3.47
		HL	1.63	0.40	n.d.	2.84	1.97	0.23	0.12	n.d.	n.d.	n.d.	n.d.	7.19
	*Dunaliella* sp.	LL	2.57	0.44	n.d.	9.21	1.12	0.27	0.35	n.d.	n.d.	n.d.	n.d.	13.96
		HL	3.74	0.57	n.d.	11.67	1.87	0.35	0.59	n.d.	n.d.	n.d.	n.d.	18.79
	*Picochlorum* sp.	LL	1.26	0.23	n.d.	4.51	0.13	0.02	0.05	n.d.	n.d.	n.d.	n.d.	6.20
		HL	0.54	0.08	n.d.	2.03	0.05	0.01	0.04	n.d.	n.d.	n.d.	n.d.	2.75
	*Schizochlamydella* sp.	LL	0.08	0.01	n.d.	0.31	0.02	0.01	0.01	n.d.	n.d.	n.d.	n.d.	0.44
		HL	0.12	0.02	n.d.	0.91	0.02	0.01	0.02	n.d.	n.d.	n.d.	n.d.	1.10
Bacillaryophyta	*Nitzschia* sp. *A*	LL	n.d.	n.d.	n.d.	0.07	0.07	n.d.	n.d.	2.35	n.d.	0.02	0.26	2.77
		HL	n.d.	n.d.	n.d.	0.06	0.06	n.d.	n.d.	1.44	n.d.	0.02	0.26	1.84
	*Nitzschia* sp. *B*	LL	n.d.	n.d.	n.d.	n.d.	0.32	n.d.	n.d.	6.25	n.d.	0.43	0.68	7.68
		HL	n.d.	n.d.	n.d.	n.d.	0.27	n.d.	n.d.	5.16	n.d.	0.37	0.56	6.36
	*Thalassiosira weissflogi*	LL	n.d.	n.d.	n.d.	n.d.	0.36	n.d.	n.d.	3.76	n.d.	0.60	0.91	5.63
		HL	n.d.	n.d.	n.d.	n.d.	0.33	n.d.	n.d.	3.49	n.d.	0.55	0.85	5.22
	*Entomoneis punctulata*	LL	n.d.	n.d.	n.d.	n.d.	0.39	n.d.	n.d.	5.23	n.d.	0.15	0.90	6.67
		HL	n.d.	n.d.	n.d.	n.d.	0.33	n.d.	n.d.	4.34	n.d.	0.13	0.77	5.57
	*Cylindrotheca closterium*	LL	n.d.	n.d.	n.d.	n.d.	0.17	n.d.	n.d.	2.82	n.d.	0.08	0.83	3.90
		HL	n.d.	n.d.	n.d.	n.d.	0.12	n.d.	n.d.	1.61	n.d.	0.10	0.65	2.48
	*Chaetoceros* sp.	LL	n.d.	n.d.	n.d.	0.07	0.02	n.d.	n.d.	1.35	n.d.	0.21	0.07	1.72
		HL	n.d.	n.d.	n.d.	0.08	0.11	n.d.	n.d.	0.78	n.d.	0.49	0.26	1.72
	*Bacillaria* sp.	LL	n.d.	n.d.	n.d.	n.d.	0.30	n.d.	n.d.	5.36	n.d.	0.21	0.72	6.59

n.d.: Not detected.

**Table 4:** Pearson correlation test between major carotenoid content and antioxidant activities measured with oxygen radical absorbance capacity (ORAC) and thiobabituric acid reactive substances (TBARS) assays.

	ORAC Assay		TBARS Assay	
	Correlation Coefficient	R^2^	Correlation Coefficient	R^2^
total carotenoids	0.71 **	0.51	−0.12 ^ns^	-
total xanthophylls	0.71 **	0.51	−0.10 ^ns^	-
lutein	0.78 **	0.60	−0.34 ^ns^	-
zeaxanthin	0.70 *	0.48	−0.18 ^ns^	-
fucoxanthin	0.60 *	0.35	−0.24 ^ns^	-
β-Carotene	0.36 ^ns^	-	−0.30 ^ns^	-

ns: Non significant, *: *p* < 0.05, and **: *p* < 0.01.

## Supplementary Materials

The following are available online at https://www.mdpi.com/1660-3397/18/2/122/s1, Figure S1: Carotenoids distribution among the twelve microalgae species studied.
