# Supplementary figures and images for: Impact of Light Intensity on Antioxidant Activity of Tropical Microalgae
Source: Mar Drugs. 2020 Feb 18;18(2):122. doi: 10.3390/md18020122 (PMC7073765; doi:10.3390/md18020122)

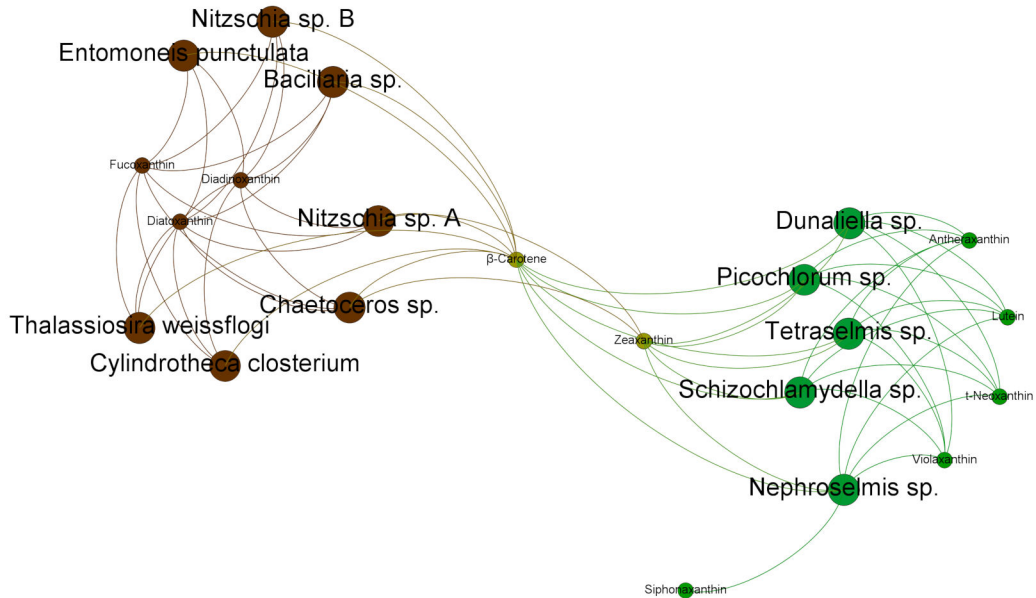

Supplement: Supplementary file 1 [file marinedrugs-18-00122-s001.pdf]
